# Abnormal ECG detection based on an adversarial autoencoder

**DOI:** 10.3389/fphys.2022.961724

**Published:** 2022-09-02

**Authors:** Lianfeng Shan, Yu Li, Hua Jiang, Peng Zhou, Jing Niu, Ran Liu, Yuanyuan Wei, Jiao Peng, Huizhen Yu, Xianzheng Sha, Shijie Chang

**Affiliations:** ^1^ Department of Intelligent Computation, School of Intelligent Medicine, China Medical University, Shenyang, China; ^2^ Division of Biomedical Engineering, School of Intelligent Medicine, China Medical University, Shenyang, China; ^3^ Department of Cardiovascular Medicine, The First Affiliated Hospital of China Medical University, Shenyang, China

**Keywords:** outlier detection (OD), autoencoder (AE), generative adversarial network (GANs), ECG, temporal convolutional network (TCN)

## Abstract

Automatic detection and alarm of abnormal electrocardiogram (ECG) events play an important role in an ECG monitor system; however, popular classification models based on supervised learning fail to detect abnormal ECG effectively. Thus, we propose an ECG anomaly detection framework (ECG-AAE) based on an adversarial autoencoder and temporal convolutional network (TCN) which consists of three modules (autoencoder, discriminator, and outlier detector). The ECG-AAE framework is trained only with normal ECG data. Normal ECG signals could be mapped into latent feature space and then reconstructed as the original ECG signal back in our model, while abnormal ECG signals could not. Here, the TCN is employed to extract features of normal ECG data. Then, our model is evaluated on an MIT-BIH arrhythmia dataset and CMUH dataset, with an accuracy, precision, recall, F1-score, and AUC of 0.9673, 0.9854, 0.9486, 0.9666, and 0.9672 and of 0.9358, 0.9816, 0.8882, 0.9325, and 0.9358, respectively. The result indicates that the ECG-AAE can detect abnormal ECG efficiently, with its performance better than other popular outlier detection methods.

## 1 Introduction

Cardiovascular diseases (CVDs) are leading causes of human death (R.L. Sacco et al., 2016), and ECG is an important method of diagnosing CVDs. Earlier detection of abnormal ECG is the key step in prevention, identification, and diagnosis of CVDs. Portable ECG could detect sudden abnormal ECG events in the early stage (Dong and Zhu, 2004) and activate warning; it is expected to reduce the mortality rate. Therefore, automatic identification of abnormal ECG events is the first important part of an ECG monitoring system.

Currently, popular artificial intelligence (AI) ECG diagnosis methods, including machine learning (feature extraction and classifiers) and deep networks, always detect abnormal ECG events using classification models. In machine learning, self-organizing map (SOM) (M.R. Risk et al., 1997), C-means clustering (Özbay et al., 2011), etc. are some of the successful machine learning methods for ECG classification. They extract features, such as wavelet coefficients (P. De Chazal et al., 2000) and autoregressive coefficients (N. Srinivasan et al., 2002), as ECG presentation. Other research studies focus on deep learning for ECG analysis, including convolutional neural networks (CNNs) (U.R. Acharya et al., 2017) and recurrent neural networks (RNNs) (H.M. Lynn et al., 2019). Xia used a deep convolutional neural network (DCNN) (Xia et al., 2018) for atrial fibrillation detection from short ECG signals (<5s) without any designed feature extraction procedure. Martin used long a short-term memory network (LSTM) (H. Martin et al., 2021) to detect myocardial infarction from a single lead ECG signal. Onan, 2020 proposed a CNN-LSTM framework for sentiment analysis of product review on Twitter. Onan and Tocoglu (2021) proposed a three-layer stacked bidirectional LSTM architecture to identify sarcastic text documents. Deep ECG (C. Li et al., 2021) takes ECG images as inputs and performs arrhythmia classification using the DCNN and transfer learning. Furthermore, a new method combining a recurrence plot (RP) and deep learning in two stages (B.M. Mathunjwa et al., 2021) is proposed to detect arrhythmias.

These aforementioned supervised learning ECG interpreting methods have achieved sound performance in previous studies. But these classification frameworks require the dataset to include all types of heart disease data with accurate manual annotation by professional doctors. The clinical ECG data are always imbalanced with fewer abnormal ECG samples, which makes it difficult to establish an effective classification model. Moreover, it is difficult to establish a large dataset including all types of abnormal ECG for clinical purposes in practice. Therefore, the sensitivity and specificity of abnormal ECG detection cannot meet clinical requirements (O. Faust et al., 2018). An outlier detection method (G. Pang et al., 2021) is more suitable for abnormal ECG in an early warning system, only based on normal data in clinical applications.

The outlier detection methods are unsupervised machine learning methods including clustering and semi-supervision including deep learning. In unsupervised methods, statistical methods usually focus on modeling the distribution of normal categories by learning the parameters of the probability model, to identify abnormal categories as outliers with low probability. The distance-based outlier detection methods assume that the normal categories are close to each other, while the abnormal samples are far away from the normal ones. Thus, outliers could be identified by calculating the distance between the abnormal and normal samples. Bin Yao and Hutchison (2014) proposed a density-based local outlier detection method (LOF) for uncertain data. H. Shibuya and Maeda (2016) developed an anomaly detection method based on multidimensional time-series sensor data and using normal state models. Principal component analysis (Li and Wen, 2014) could be used for linear models; and the Gaussian mixture model (GMM) (Dai and Gao, 2013), isolation forest (F.T. Liu et al., 2008), and one-class support vector machine (OC-SVM) (B. Schölkopf et al., 2000) are used in actual outlier detection applications. But these machine learning algorithms often require the manual design of effective features.

Performance of an outlier detection method based on deep learning has been proved well, including Auto-Encoder (Zhou and Paffenroth, 2017), LSTM (P. Malhotra et al., 2015), and VAE (Wang et al., 2020), and widely used in AI-aided diagnosis (T. Fernando et al., 2021) such as X-ray film, MRI, CT, and other medical images, and in the detection of EEG, ECG, and other timing signals as well. Y. Xia et al. (2015) eliminated abnormal data from noisy data by reducing reconstruction errors of the autoencoder, and applying gradients of the autoencoder to make reconstruction errors discriminatory to positive samples. By using deep neural networks (autoencoders) as feature extractors, a deep hybrid model (DHM) has been applied for outlier detection to input extracted features into traditional outlier detection algorithms, such as OC-SVM (Mo, 2016). L. Ruff et al. (2018) used deep one-class classification for end-to-end outlier detection, effectively customizing trainable targets for outlier detection to extract features. K. Li et al. (2012) proposed a transfer learning framework for detecting abnormal ECG; however, this method requires manual coding of features and relies on labeled data for all different types of abnormalities. Due to diversity of diseases and different waveforms collected from different abnormal diseases, such data are not easy to obtain. Time series outlier detection technology is also used in ECG signal processing; Lemos and Tierra-Criollo, 2008; Chauhan and Vig, 2015 proposed an outlier detection method based on LSTM. An abnormal condition is considered when the difference between the predicted value of LSTM and normal value exceeds a given threshold. Latif et al. (2018) used a recurrent neural network (RNN) to detect abnormal heartbeats in the PCG signal detection of the heart sound, which needs a large amount of calculation. K. Wang et al. (2016) used an autoencoder to reconstruct normal ECG data, determine the threshold according to the reconstruction error, and finally, to detect the test set.

Recently, a GAN-based framework has been applied to outlier detection (T. Schlegl et al., 2017). The model generates new data according to the input; if the input was similar to the training data (as normal data), the output would be similar to the input, otherwise, the input would be an outlier. T. Schlegl et al. (2017) used a GAN-based model (AnoGan) to identify anomalies in medical images. However, the aforementioned methods have the problems of overfitting (C. Esteban et al., 2017) or instability (D. Li et al., 2019) when they deal with abnormal ECG detection problems.

An autoencoder is another method of simply “memorizing” the training data and reproducing them. The parameters of the intermediate hidden layer would completely fit the training set, and the content of its memory will be completely output at the time of the output, resulting in identity mapping of the neural network and data overfitting. Problems such as instability and poor controllability occur with the latent model based on the GAN method.

In this study, we proposed a novel method named ECG-AAE for detecting abnormal ECG events, based on an adversarial autoencoder and TCN (L. Sun et al., 2015). It consists of three parts: 1) an autoencoder, 2) a discriminator, and 3) an outlier detector. Our method was evaluated on the MIT-BIH and our CMUH datasets and compared with several other popular outlier detection methods.

## 2 Materials

### 2.1 Electrocardiogram datasets

1) Massachusetts Institute of Technology Arrhythmia Dataset (MIT-BIH). The dataset consists of 48 double-lead ECG recordings from 47 subjects; each set lasts 30 min at a sample rate of 360 Hz, with approximately 110,000 beats. A set of beat labels is equipped at the peak of R.

2) A CMUH dataset supported by the First Affiliated Hospital of China Medical University. The dataset contains 12-lead ECG records of inpatients in the First Affiliated Hospital of China Medical University from January 2013 to December 2017, with a sampling rate of 560 Hz.

Two or three cardiologists annotated all heartbeats for both datasets independently. Only lead II ECG signals are used in this study.

### 2.2 Data preprocessing

**FIGURE 1:**
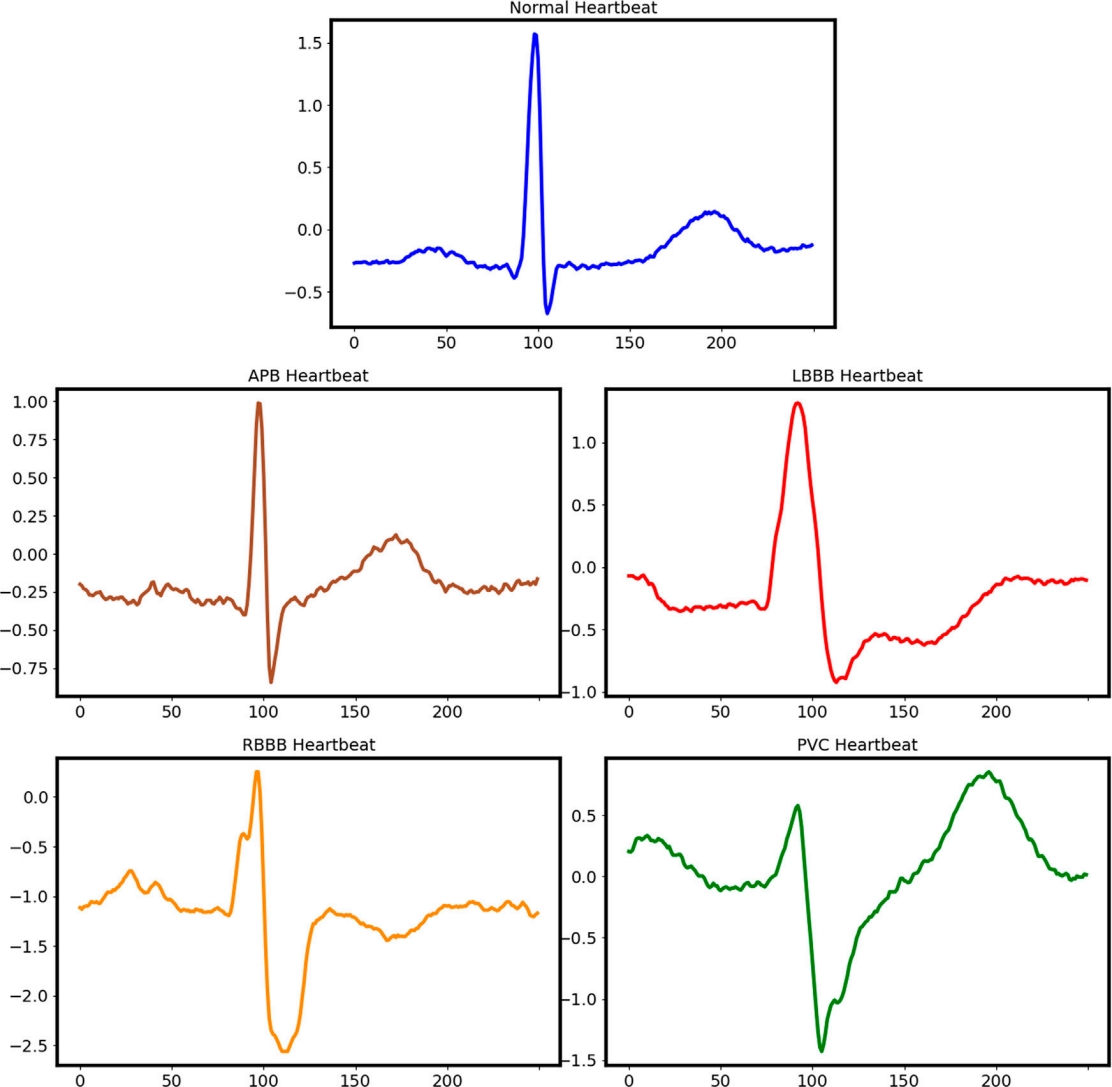
Typical sketch of normal and abnormal heartbeats.

A total of four types of arrhythmia and normal beats are selected from datasets: right bundle branch block (R), left bundle branch block (L), atrial premature beat (A), ventricular premature beat (V), and normal sinus rhythm (N). ECG signals are split into single heartbeats which are normalized to a range of [−1, 1] for network training. Five typical heartbeats are shown in Figure 1.

### 2.3 MIT-BIH dataset

In this study, 45 lead II signal records are selected from the MIT-BIH dataset (records 102, 104, and 114 were excluded, as they do not include the lead II data or the type of heart disease in our experiments). Wavelet transform is used to reduce noise and baseline drift (Alfaouri and Daqrouq, 2008). Then, the ECG data are split into single heartbeats using the marked R peak location. A total of 250 points (100 points before the R peak and 150 points after the R peak) are included in a heartbeat.

### 2.4 CMUH dataset

ECG data of 44,173 people from the CMUH dataset have been selected for this study. Data are resampled at 360 Hz to maintain consistency with MIT-BIH data. The beat segmentation method is the same as the one mentioned previously.

For each dataset, 10,000 normal ECG data are randomly selected as the training set, and 5,000 normal ECG data and 5,000 abnormal ECG data are randomly selected as the test set, as shown in Table 1.

**TABLE 1:** Number of heartbeats involved in each dataset and the division of datasets.

Dataset	Type	Type of heartbeats	Number of heartbeats	Number of cases	Sample size	Number of training set	Number of test set
MIT-BIH	Normal	N	74,962	40	15,000	10,000	5,000
	Abnormal	A	2,545	—	5,000	0	5,000
		L	8,068	—			
		R	7,254	—			
		V	7,034	—			
		Total	99,863	47	20,000	10,000	10,000
CMUH	Normal	N	20,000	20,000	15,000	10,000	5,000
	Abnormal	A	6,811	6,811	5,000	0	5,000
		L	1,247	1,247			
		R	8,268	8,268			
		V	7,847	7,847			
		Total	44,173	44,173	20,000	10,000	10,000

## 3 Methods

### 3.1 ECG-AAE framework

**FIGURE 2:**
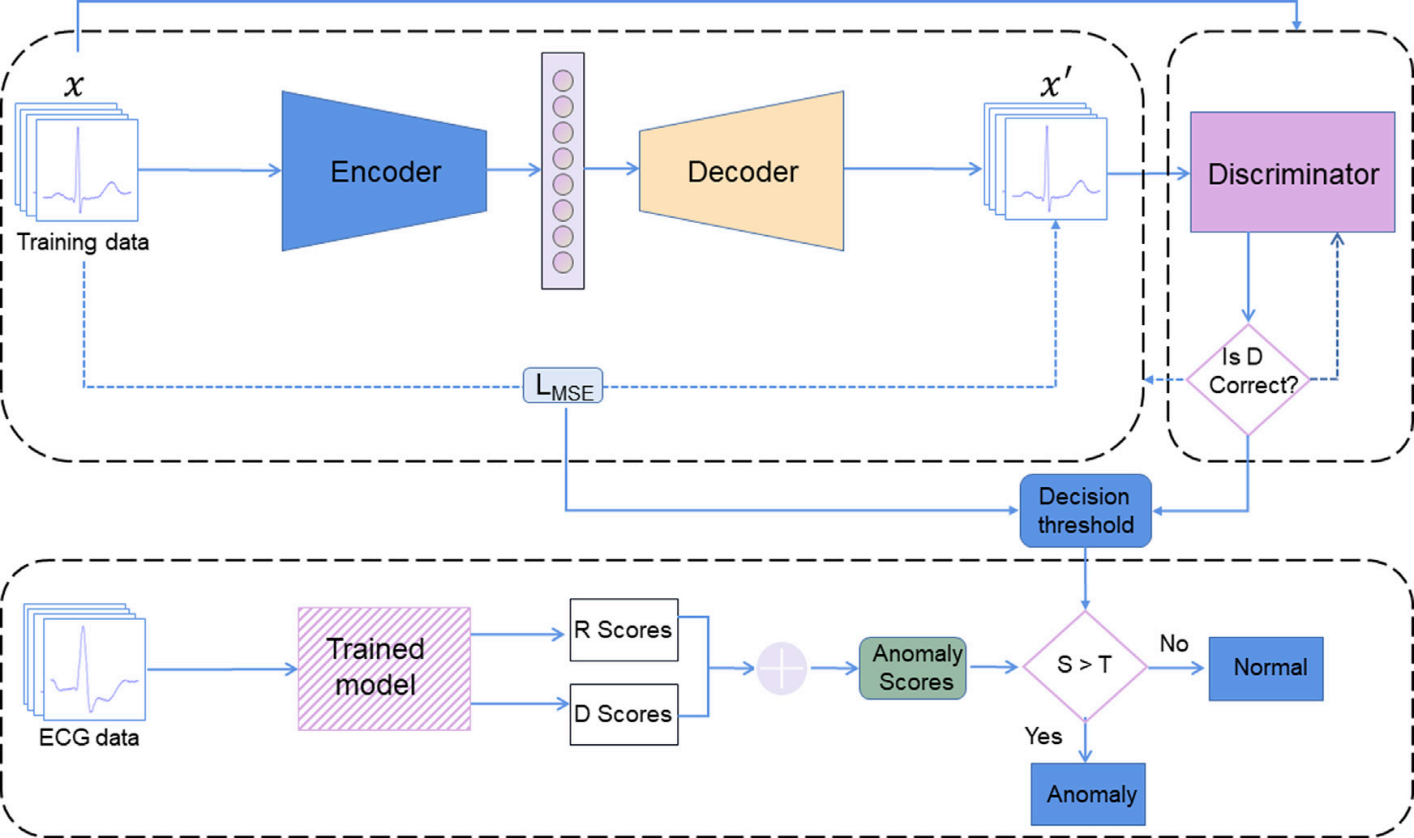
ECG-AAE framework consists of three parts: (1) an autoencoder, (2) a discriminator, and (3) an outlier detector. Here, R score is the reconstruction error score; D score is the discrimination score; S is the anomaly scores (the sum of R score and D score). T is the threshold of outlier data.

The ECG-AAE framework consists of three parts: 1) an autoencoder, 2) a discriminator, and 3) an outlier detector, as shown in Figure 2. The autoencoder tries to minimize reconstruction errors to generate ECG signals similar to input signals. The discriminator uses reconstructed and original data as the input, and is trained to distinguish normal data from reconstructed data. Both the autoencoder and discriminator update simultaneously to improve the reconstruction performance of the autoencoder.

Finally, the combination of reconstruction errors and discriminant scores (probability output of discriminator) is used to evaluate normal ECG. Test data are mapped back to potential space, and loss between reconstructed test samples and actual test samples has been applied to calculate the corresponding reconstruction loss.

A detailed network of the ECG-AAE is shown in Table 2. The encoder is composed of three TCN blocks, three MaxPooling1D layers, a flatten layer, and a dense layer. The decoder is composed of a dense layer, three TCN blocks, three UpSampling1D layers, and a Conv1D layer. The discriminator is composed of three TCN blocks, three MaxPooling1D layers, a flatten layer, and two dense layers. The activation function for the last dense layer is sigmoid. A large discriminator can make the data overflow easily, while a shallow autoencoder cannot generate enough real data to defeat the discriminator. A small number of hidden units is chosen as the starting point, and the number of hidden units has been gradually increased in each successive layer, which is effective for the training of the model in this study. Also, three TCN blocks are used in the encoder, decoder, and discriminator.

**TABLE 2:** Detailed overview of the proposed ECG-AAE model.

Modules	Layers	Types	Activation function	Output shapes	Kernel size	No. of filters
Encoder	0	Input	—	250 × 1	—	—
	1	TCN block	ReLU	250 × 32	9	32
	2	MaxPooling1D	—	50 × 32	—	—
	3	TCN block	ReLU	50 × 16	9	16
	4	MaxPooling1D	—	10 × 16	—	—
	5	TCN block	ReLU	10 × 8	9	8
	6	MaxPooling1D	—	2 × 8	—	—
	7	Flatten	—	16	—	—
	8	Dense	ReLU -	8	—	—
Decoder	0	Input	—	8	—	—
	1	Dense	ReLU	16	—	—
	2	Reshape	—	2 × 8	—	—
	3	UpSampling1D	—	10 × 8	—	—
	4	TCN block	ReLU	10 × 8	9	8
	5	UpSampling1D	—	50 × 16	—	—
	6	TCN block	ReLU	50 × 16	9	16
	7	UpSampling1D	—	250 × 16	—	—
	8	TCN block	ReLU	250 × 32	9	32
	9	Conv1D	ReLU	250 × 1	9	1
Discriminator	0	Input	—	250 × 1	—	—
	1	TCN block	ReLU	250 × 32	9	32
	2	MaxPooling1D	—	50 × 32	—	—
	3	TCN block	ReLU	50 × 16	9	16
	4	MaxPooling1D	—	10 × 16	—	—
	5	TCN block	ReLU	10 × 8	9	8
	6	MaxPooling1D	—	2 × 8	—	—
	7	Flatten	—	16	—	—
	8	Dense	ReLU	8	—	—
	9	Dense	sigmoid	1		

In this study, stochastic gradient descent (Adam) (Kingma and Ba, 2015) is adopted to conduct alternating update training for each lost component, and parameters of the network model are obtained through training and learning.

### 3.2 Temporal convolutional network

**FIGURE 3:**
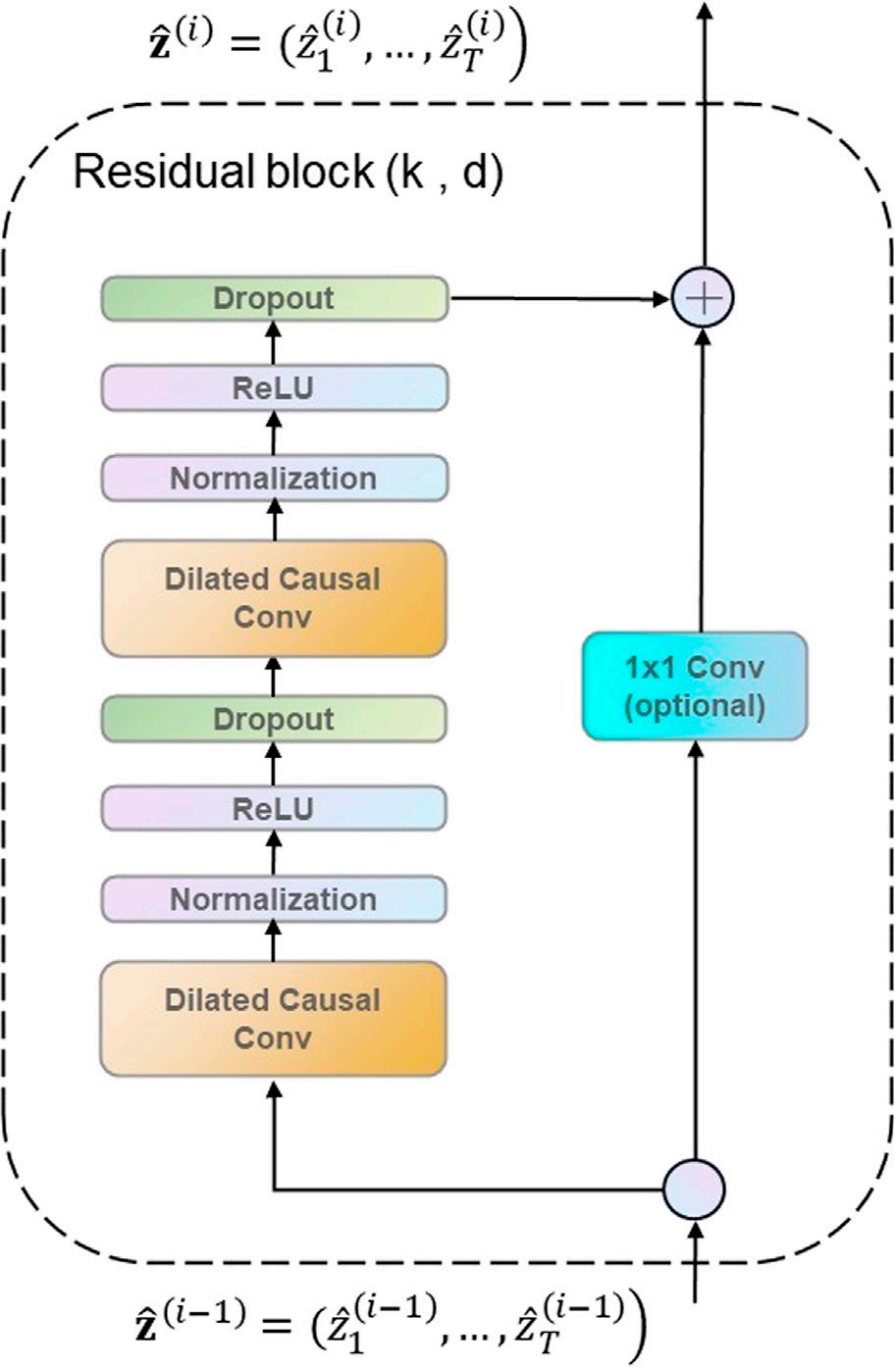
Residual block.

Atemporal convolutional network (TCN) (L. Sun et al., 2015) could capture long-term dependence in an ECG sequence more effectively. A TCN block is superimposed by two causal convolution layers with the same expansion factor, followed by normalization, ReLU, and dropout layers, as shown in Figure 3.

The TCN is used to extract features of ECG time series data. The TCN module has shown competitiveness in many sequence-related modeling tasks (W. Zhao et al., 2019). It can capture dependencies in sequences more effectively than recurrent neural networks (Graves et al., 2013; Z. Huang et al., 2015; J. Chung et al., 2014). The TCN convolution kernel is shared in the same layer, with lower requirement memory.

**FIGURE 4:**
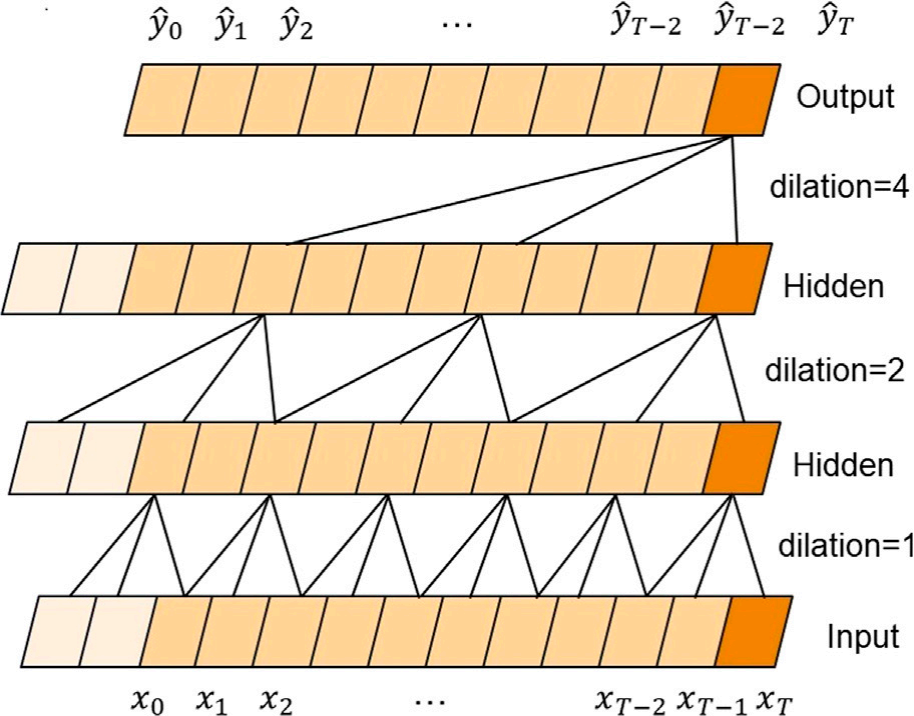
Stacked convolutional layers in the TCN.

The TCN is mainly composed of dilated causal convolution. Figure 4 shows a simple structure of TCNs, where x_i_ represents the characteristics of the *i*th moment. Expanded convolution enables input interval sampling during convolution, and the sampling rate is controlled by d. The parameter *d* = 1 in the bottom layer means that every point is sampled as input, and *d* = 2 in the middle layer means that every two points are sampled as input. Generally, the higher the level, the larger will be the value of d used, with the size of the effective window of dilated convolution increasing exponentially with the number of levels. Convolution networks can obtain a larger receptive field with fewer layers.

The TCN uses a residual block structure which is similar to that in ResNet to solve problems such as a deeper network structure causing gradient disappearance, to make the model more generic. A residual block superimposes multiple causal convolutional layers with the same expansion factor, followed by normalization, ReLU, and dropout. In this study, a residual block containing two layers of convolution and nonlinear mapping is constructed, and normalization and dropout to each layer are added to regularize the network, as shown in Figure 3.

### 3.3 Autoencoder module

An autoencoder module consists of three parts: an encoder, a hidden layer, and a decoder. Only normal ECG data are used for training. First, input data x are compressed and encoded into the hidden layer data, and then hidden layer data are decoded to obtain reconstructed ECG data ${\text{X}}^{\prime }$ . The loss function during training is the reconstruction error between input data x and output data X':

$$\text{Loss}\left(\text{X},{\text{X}}^{\prime }\right)={\left\|\text{X}-{\text{X}}^{\prime }\right\|}^{2}$$ (1)

The encoder and decoder are optimized to minimize reconstruction errors of normal ECG using training data X .

The activation functions of the encoder and decoded neural networks are shown as follows:

Z=δ(WX+b) (2)

$${\text{X}}^{\prime }={\text{δ}}^{\prime }\left({\text{W}}^{\prime }\text{Z}+{\text{b}}^{\prime }\right)$$ (3)

where, δ and δ' are non-linear exciting functions, and W, b, W', and b' are weights and offsets of linear transformations.

Minimizing the loss function to optimize the parameters in the encoder and decoder is equivalent to a nonlinear optimization problem:

$${\text{min}}_{\text{δ},\text{w},\text{b}}\text{Loss}\left(\text{X},{\text{X}}^{\prime }\right)={\left\|\text{X}-{\text{δ}}^{\prime }\left(\text{δ}\left(\text{WX}+\text{b}\right)\right)+{\text{b}}^{\prime }\right\|}^{2}$$ (4)

### 3.4 Discriminator module

The discriminator (D) is to distinguish reconstructed ECG data ${\text{X}}^{\prime }$ generated by the autoencoder (AE) from real data X during the training process, and to make reconstructed data similar to the input data. Thus, the autoencoder tries to minimize the reconstruction error, while the discriminator tries to maximize it. During training, the two modules optimize themselves and improve refactoring and discrimination. The autoencoder is trained to minimize the difference between reconstructed and input samples, and the discriminator is trained to maximize confidence in discriminating the difference between reconstructed and real samples. After training, the discriminator assigns correct labels to real and fake ECG data as sensitively as possible, while the autoencoder generates real ECG data as much as possible to deceive the discriminator, and the two reach a balance (D. Li et al., 2019). The conditional autoencoder and discriminator are trained following a two-player minimax game:

VAE Dminmax(D,AE)=εx∼Pdata(X)[logD(x)]+εx∼Pz(Z)[log(1−D(AE))] (5)

### 3.5 Outlier detection module

The combination of reconstruction errors and discriminant scores is used to define the abnormal score. Reconstruction loss R(x) makes a higher score on abnormal ECG data and a lower score on normal ECG data. The discrimination score D(x) produces lower scores on abnormal ECG data and higher scores on normal ECG data.

Therefore, the anomaly score a(x) formula is expressed as

$$\text{a}\left(\text{x}\right)=\left(1-\text{λ}\right)\text{R}\left(\text{x}\right)+\text{λ}\frac{1}{\text{D}\left(\text{x}\right)}$$ (6)

λ = 0, according to our experience. The threshold is decided following one standard deviation above the mean. ECG data with a(x) greater than the threshold are abnormal.

## 4 Results

### 4.1 Evaluation indexes

Accuracy (ACC), precision (Pre), recall (Rec), F1-score (F_1_), and AUC value (area under the ROC curve) are used to evaluate the performance of our ECG-AAE and compare it with other methods. In the confusion matrix, abnormal ECG is defined as positive, normal ECG is defined as negative, and true positive (TP), true negative (TN), false positive (FP), and false negative (FN) are calculated.

$$\text{ACC}=\frac{\text{TP}+\text{TN}}{\text{TP}+\text{TN}+\text{FP}+\text{FN}}$$

$$\text{Pre}=\frac{\text{TP}}{\text{TP}+\text{FP}}$$

$$\text{Rec}=\frac{\text{TP}}{\text{TP}+\text{FN}}$$

$${\text{F}}_{1}=2\times \frac{\text{Pre}\times \text{Rec}}{\text{Pre}+\text{Rec}}$$

In clinical practice, the precision rate represents the proportion of patients with true ECG abnormalities, while recall rate represents the proportion of patients with true ECG abnormalities. The high-precision detection model could prevent misdiagnosis, while the detection model with a high recall rate could avoid missed diagnosis. The F1-score is a weighted harmonic average of the recall rate and accuracy rate; the F1-score and AUC value are used as the main indicators to measure the performance of outlier detection in this study.

The experiment was implemented on a workstation (Dell T7600, Xeron 2,650 × 2, 256 GB RAM, 1080Ti×2), with Linux 18.04, Python 3.6, Keras 2.3.1, and TensorFlow 2.0.

#### 4.1.1 Experiment 1

**FIGURE 5:**
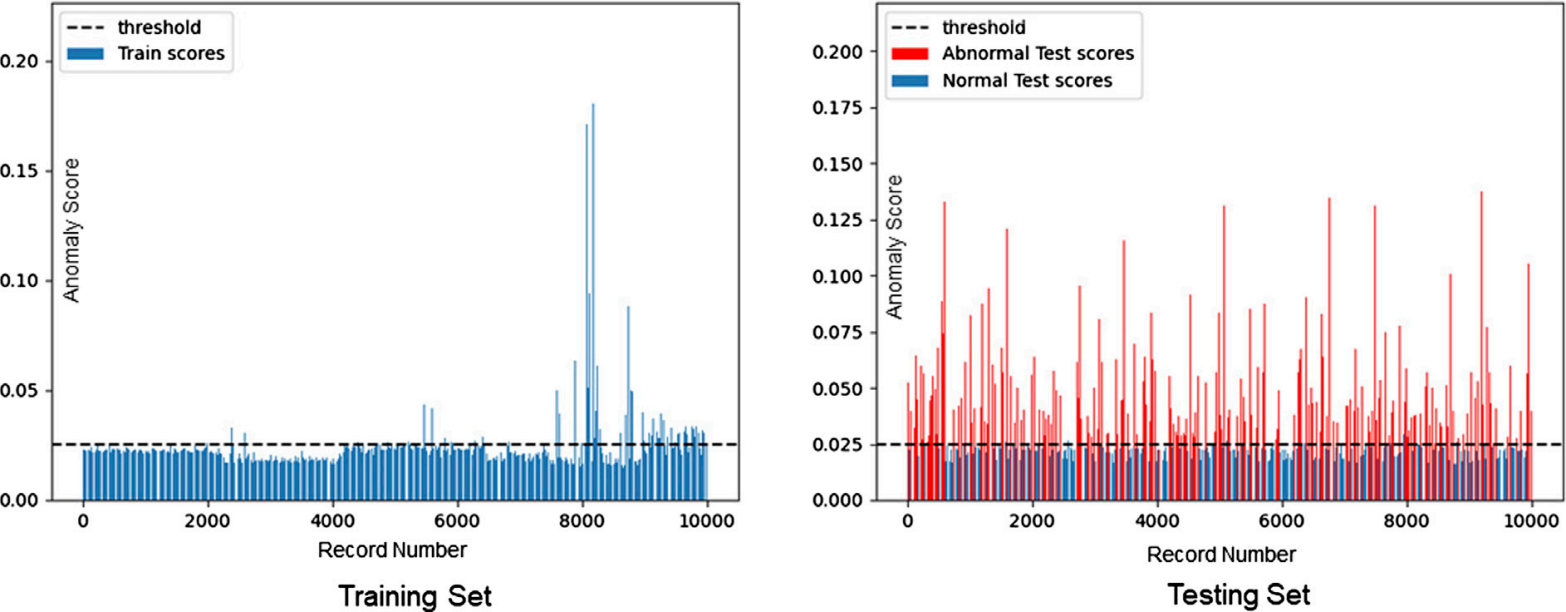
Distribution of anomaly scores of the MIT-BIH training set (T = 0.025).

**FIGURE 6:**
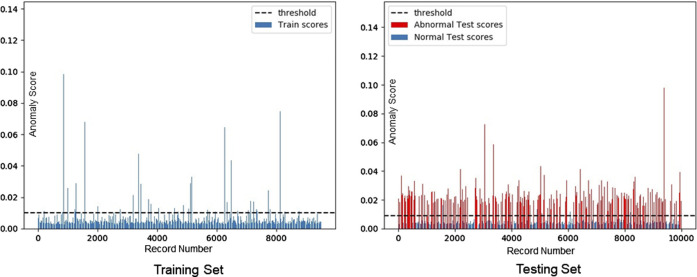
Distribution of anomaly scores of the CMUH training set (T = 0.01).

Both MIT-BIH and CMUH datasets have been used to verify the performance of our framework. The threshold value is selected as one standard deviation above the mean according to the abnormal score in the training set. T values of MIT-BIH and CMUH datasets can be obtained as 0.025 and 0.01, respectively. When the training set includes normal data only, its abnormal scores are within the range of the threshold T (Figures 5A, 6A), while, in the test dataset including both normal and abnormal ECG data, the abnormal scores are less than the threshold T for normal ECG data, but are greater than the threshold T (Figures 5B, 6B) for abnormal ECG data.

**FIGURE 7:**
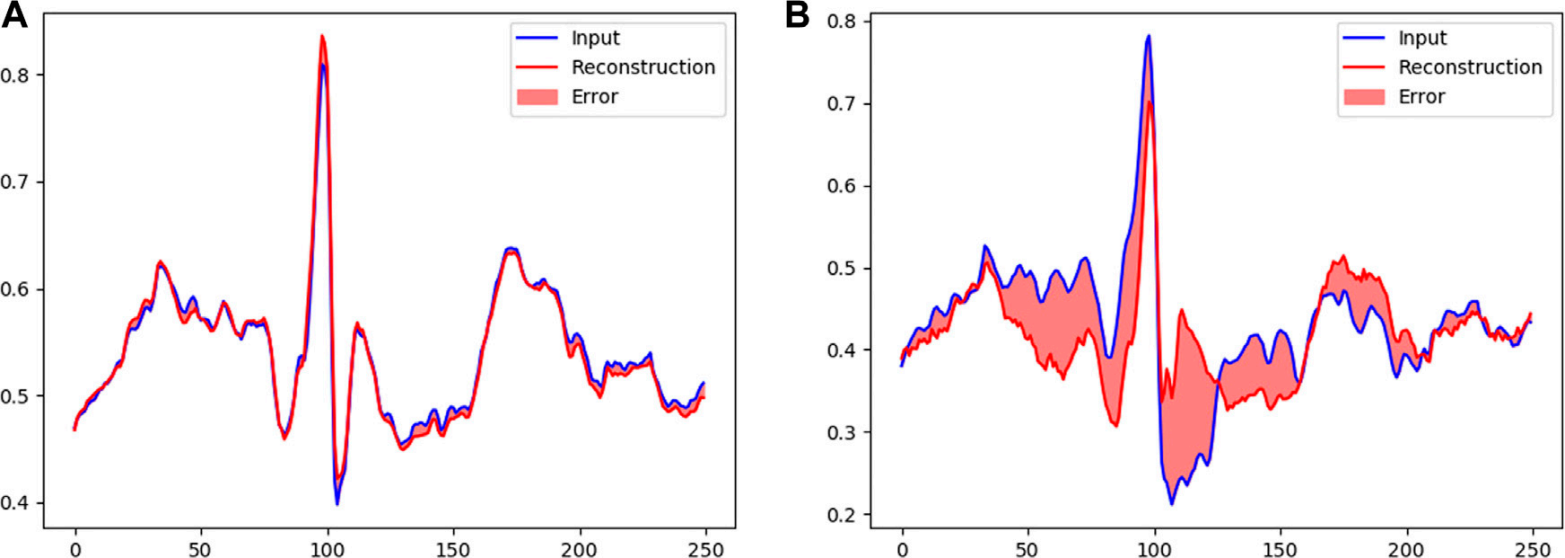
Reconstruction of ECG data: **(A)** normal and **(B)** abnormal.

An example of normal and abnormal ECG data reconstructed by our model is shown in Figure 7. For normal ECG data, reconstructed data are continuous, and the shape of the reconstructed waveform is basically the same as the input one, with an error range of 0.0063 ± 0.0098. For abnormal ECG data, the shape of the reconstructed waveform differs greatly from that of the input waveform. Although the reconstructed data are continuous, the error range reaches 0.0289 ± 0.0264.

**FIGURE 8:**
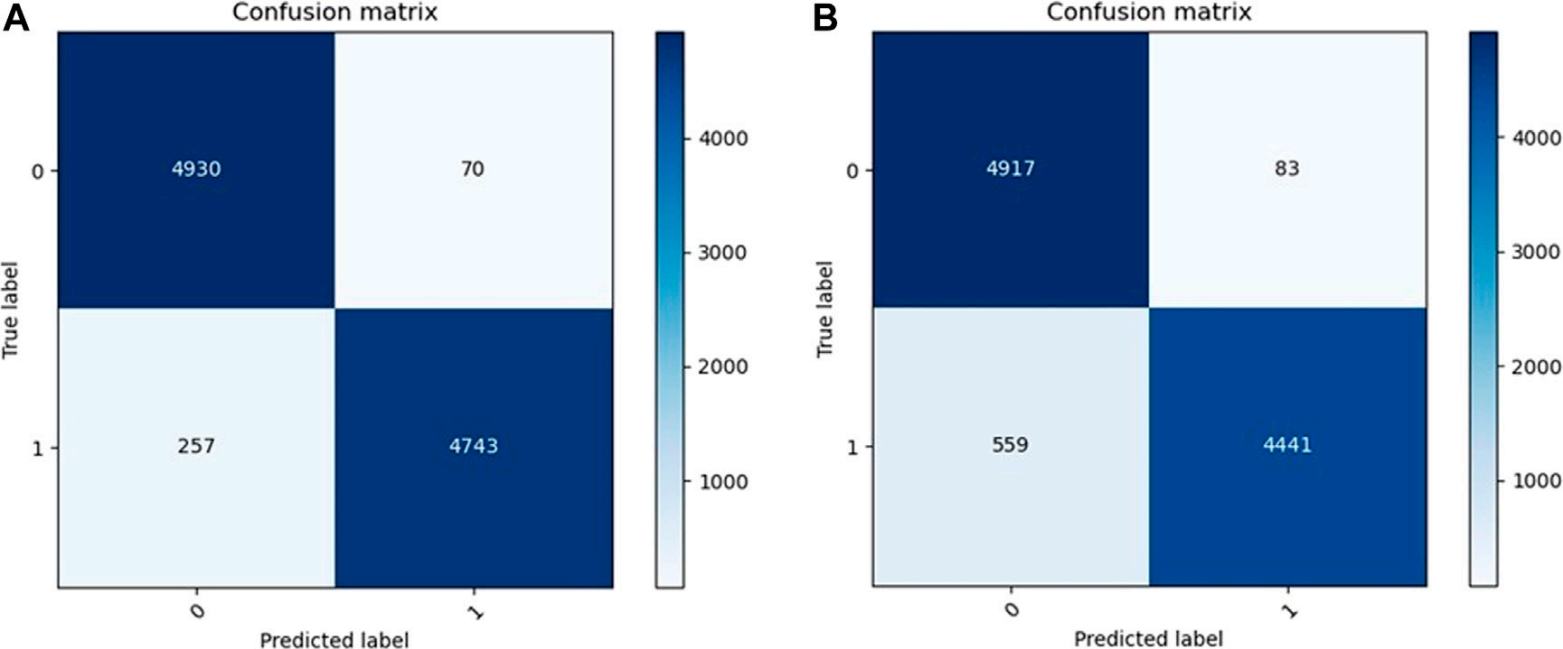
Confusion matrix of **(A)** MIT-BIH dataset and **(B)** CMUH dataset (1 = normal 0 = abnormal).

The confusion matrixes of detection results are shown in Figure 8. In the MIT-BIH dataset, 4,930 abnormal ECGs were detected, and 257 normal ECGs were predicted as abnormal. In our CMUH dataset, 4,917 abnormal ECG data were detected, and 559 normal ECGs were predicted as abnormal. The accuracy, recall, F1 score, and AUC of our model are 0.9673, 0.9854, 0.9486, 0.9666, and 0.9672, and 0.9358, 0.9816, 0.8882, 0.9325, and 0.9358, respectively.

#### 4.1.2 Experiment 2

Our method was compared with 13 popular outlier detection methods using MIT datasets, as shown in Table 3. Among the five evaluation indicators, our model achieves the highest score of 0.9673 in accuracy. DAGMM achieves the highest score of 0.9992 in precision, but its recall is 0.5304. This shows that DAGMM tries to predict the sample as a positive sample when it is “more certain,” but misses many unsure positive samples due to its excessive conservativeness. The AE achieves the highest recall score of 0.9902, but the precision is 0.8829, indicating that the AE produces more false positives. The ECG-AAE model achieves the highest scores of 0.9673, 0.9666, and 0.9672 in accuracy, F1-score, and AUC value, respectively, better than other models.

**TABLE 3:** Average classification performance for different methods on the MIT-BIH dataset.

Methods	Acc ±SD	Pre ±SD	Rec ±SD	F1-score ± SD	AUC ±SD
OURS	**0.9673 ± 0.0005**	0.9854 ± 0.0003	0.9486 ± 0.0001	**0.9666 ± 0.0014**	**0.9672 ± 0.0015**
AnoGAN (Schlegl et al.)	0.9257 ± 0.0101	0.8829 ± 0.0167	0.9876 ± 0.0027	0.9323 ± 0.0085	0.9283 ± 0.0101
AE (K.Wang et al.)	0.9282 ± 0.0180	0.8733 ± 0.2042	**0.9902 ± 0.0233**	0.9281 ± 0.1490	0.9233 ± 0.0049
VAE (X.Wang et al.)	0.8048 ± 0.0028	0.7196 ± 0.0029	0.9874 ± 0.0002	0.8325 ± 0.0157	0.8013 ± 0.0028
Stack LSTM (Chauhan et al.)	0.8875 ± 0.0017	0.8313 ± 0.0021	0.9740 ± 0.0007	0.8970 ± 0.0019	0.8882 ± 0.0052
GRU (Cowton et al.)	0.8764 ± 0.0040	0.8128 ± 0.0064	0.9746 ± 0.0017	0.8864 ± 0.0031	0.8751 ± 0.0040
RNN (Latif et al.)	0.8568 ± 0.0031	0.7826 ± 0.0040	0.9798 ± 0.0003	0.8702 ± 0.0024	0.8538 ± 0.0031
DEEP-SVDD (Ruff et al.)	0.8039 ± 0.0035	0.7221 ± 0.0037	0.8342 ± 0.0002	0.8342 ± 0.0025	0.8037 ± 0.0033
AE + OCSVM (Mo et al.)	0.8624 ± 0.0036	0.7965 ± 0.0046	0.9788 ± 0.0003	0.8783 ± 0.0029	0.8644 ± 0.0050
DAGMM (Song et al.)	0.7646 ± 0.0007	**0.9992 ± 0.0008**	0.5304 ± 0.0004	0.6930 ± 0.0019	0.7650 ± 0.0019
GMM (Dai et al.)	0.6462 ± 0.0463	0.9986 ± 0.1603	0.2924 ± 0.0068	0.4524 ± 0.0274	0.6460 ± 0.0042
OCSVM (Schölkopf et al.)	0.8376 ± 0.0009	0.9982 ± 0.0006	0.6760 ± 0.0005	0.8061 ± 0.0018	0.8374 ± 0.0019
iForest (Liu et al.)	0.6521 ± 0.0106	0.9987 ± 0.2119	0.3046 ± 0.3468	0.4668 ± 0.1334	0.6521 ± 0.0106
LOF (Bin Yao et al.)	0.5050 ± 0.0006	0.5027 ± 0.0007	0.9170 ± 0.0025	0.6494 ± 0.0018	0.5050 ± 0.0020

SD, standard deviation.

The bold values mean maximum.

#### 4.1.3 Experiment 3

We further verify the robustness and generalization of the model with our CMUH dataset, as shown in Table 4.

**TABLE 4:** Average classification performance for different methods on the CMUH dataset.

Methods	Acc ± SD	Pre ± SD	Rec ± SD	F1-score ± SD	AUC ± SD
OURS	**0.9358 ± 0.0004**	0.9816 ± 0.0002	0.8882 ± 0.0010	**0.9325 ± 0.0008**	**0.9358 ± 0.00010**
AnoGAN (Schlegl et al.)	0.8985 ± 0.0092	0.8396 ± 0.0128	0.9852 ± 0.0018	0.9066 ± 0.0078	0.8985 ± 0.0092
AE (K.Wang et al.)	0.9103 ± 0.0181	0.8504 ± 0.0253	**0.9946 ± 0.0012**	0.9169 ± 0.0148	0.9098 ± 0.0181
VAE (X.Wang et al.)	0.7744 ± 0.0040	0.6885 ± 0.0039	0.9910 ± 0.0015	0.8125 ± 0.0027	0.7713 ± 0.0041
Stack LSTM (Chauhan et al.)	0.8754 ± 0.0033	0.8097 ± 0.0051	0.9772 ± 0.0019	0.8856 ± 0.0025	0.8738 ± 0.0033
GRU (Cowton et al.)	0.8779 ± 0.0038	0.8156 ± 0.0052	0.9748 ± 0.0019	0.8881 ± 0.0030	0.8772 ± 0.0038
RNN (Latif et al.)	0.8221 ± 0.0037	0.7414 ± 0.0041	0.9860 ± 0.0021	0.8464 ± 0.0026	0.8210 ± 0.0037
DEEP-SVDD (Ruff et al.)	0.7649 ± 0.0050	0.6794 ± 0.0047	0.9908 ± 0.0017	0.8061 ± 0.0032	0.7616 ± 0.0050
AE + OCSVM (Mo et al.)	0.8245 ± 0.0030	0.7436 ± 0.0034	0.9864 ± 0.0022	0.8479 ± 0.0021	0.8231 ± 0.0030
DAGMM (Song et al.)	0.7260 ± 0.0012	0.9991 ± 0.0004	0.4520 ± 0.0024	0.6224 ± 0.0023	0.7258 ± 0.0012
GMM (Dai et al.)	0.6057 ± 0.0037	**1.0000 ± 0.0005**	0.2148 ± 0.0074	0.3536 ± 0.0101	0.6074 ± 0.0037
OCSVM (Schölkopf et al.)	0.7600 ± 0.0024	0.9985 ± 0.0010	0.5208 ± 0.0048	0.6845 ± 0.0041	0.7600 ± 0.0024
iForest (Liu et al.)	0.6303 ± 0.0043	**1.0000 ± 0.0009**	0.2606 ± 0.0086	0.4135 ± 0.0107	0.6303 ± 0.0043
LOF (Bin Yao et al.)	0.5767 ± 0.0059	**1.0000 ± 0.0010**	0.1700 ± 0.0117	0.2906 ± 0.0174	0.5850 ± 0.0059

SD, standard deviation.

The bold values mean maximum.

Our model achieves the highest scores of 0.9358, 0.9325, and 0.9358 in accuracy, F1-score, and AUC, respectively. GMM, iForest, and LOF models achieve the highest score of 1.000 in precision, but the recall was lower. The AE achieves the highest recall of 0.9946, but the F1-score and AUC value are lower.

## 5 Discussion

To solve problems that the classification model cannot effectively detect in abnormal ECGs, we propose the ECG-AAE, a framework for detecting abnormal ECG signals. Its performance is verified and compared with the AE, AnoGAN, and other 11 popular outlier detection methods on the MIT-BIH arrhythmia dataset and our CMUH dataset.

The four kinds of machine learning outlier detection algorithms with low performance scores were GMM (Dai and Gao, 2013), OCSVM (B. Schölkopf et al., 2000), iForest (F.T. Liu et al., 2008), and LOF (S.H. Bin Yao and Hutchison, 2014). Among them, GMM enjoys the best performance, whose AUC values are 0.6460 and 0.6074 on the MIT-BIH and CMUH datasets respectively; LOF, the worst model, shows AUC values are 0.5050 and 0.5850, respectively. It suggests that the machine learning methods might not be the best choice for abnormal ECG detection; they may not extract abnormal ECG effectively. Moreover, the subsequent classifiers could not fit the boundary functions in high-dimension feature space, while, the deep learning models could make ECG feature extraction more elastic to fit the nonlinear feature distribution, and finally improve the detection rate of abnormal ECG while ensuring accuracy.

Among deep learning models, generative models based on AE or GAN are better than hybrid models of machine learning and deep learning (e.g., AE + OCSVM (Mo, 2016) deep-SVDD (L. Ruff et al., 2018), DAGMM (Q. Song et al., 2018), RNN and its variants, LSTM, GRU, and other recurrent neural network models). The autoencoder Cowton et al., (K. Wang et al., 2016) encodes one-dimensional signal data into a lower dimension to learn the general distribution of data and then decodes to a higher dimension to reconstruct data. In this experiment, the AE performs well on both the MIT-BIH and CMUH datasets.

The ECG-AAE combines the autoencoder and discriminator, and it uses the autoencoder to realize reconstruction of the ECG and the discriminator to improve the generation ability of the autoencoder. The TCN could obtain ECG features at different scales with different receptive fields, which helps accurately reconstruct the normal ECG. In addition, the TCN avoids problems of gradient disappearance or gradient explosion. We use the combination of reconstruction errors and discriminant scores as the anomaly score, which effectively reduces the impact of the AE overfitting and instability of the GAN model. Compared with methods dealing with two leads or more, Liu F (2020) provided an accuracy of 97.3% in ECG anomaly detection; Thill et al. (2021 designed a temporal convolutional network autoencoder (TCN-AE) based on dilated convolutions for time series data.

Experiments 2 and 3 suggest that the CMUH dataset is about 0.3% lower than the MIT-BIH dataset on each model. The reason is that all the heartbeats in the MIT-BIH dataset are only from 48 people. These independent heartbeats are obtained through heartbeat segmentation, with very similar characteristics which are not enough for generalization, while each heartbeat in our CMUH dataset comes from a signal person, which is more in line with reality.

**FIGURE 9:**
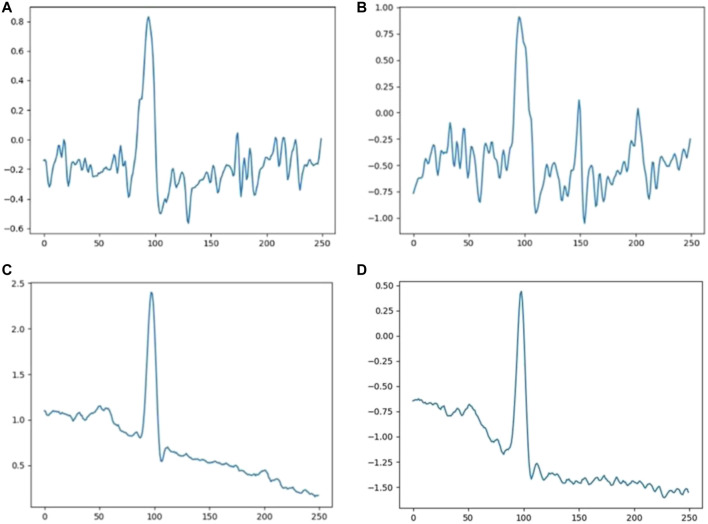
Analysis of the predication error **(A**,**B)** noise interference ECG, **(C,D)** baseline drift ECG.

False positive data are largely affected by noise interference, as shown in Figures 9A,B. At the same time, false negative data in the experiment have also been analyzed with the finding that a baseline exists in most cases, as shown in Figures 9C,D. The ECG-AAE model can tolerate noise and baseline drift of conventional static ECG, but the form of input data in these error cases is quite different from that of normal ECG data. This situation might occur when patients move in a large range. Although noise filtering and baseline drift are carried out in the data preprocessing stage, an ideal effect is not achieved on the ECG data with large variation, which leads to a false positive and negative output of the model. In clinical practice, false positives and negatives can be avoided by analyzing several continuous heartbeats, and when the several continuous heartbeats are judged to be abnormal ECGs, abnormal ECGs can be diagnosed.

## 6 Conclusion

Detection and early warning of sudden abnormal ECG is an important procedure in an ECG monitoring and alarm system. The ECG-AAE framework proposed in this study could efficiently detect abnormal ECG signals, and provide better performance on several indicators in our tests. It also suggests that outlier detection performs better than the classical classification framework in clinical practices. As far as we know, this is the first study to combine the adjournment network of abnormal ECG detection, which solves all types of abnormal ECG data and data balance problems and effectively improves the detection rate of abnormal ECG in the open set condition while ensuring accuracy.

## Data Availability

The original contributions presented in the study are included in the article/Supplementary Material; further inquiries can be directed to the corresponding author.
